# Lifestyle and reproductive health: the aetiology of ovarian cancer in Pakistan

**DOI:** 10.12688/f1000research.24866.1

**Published:** 2020-08-04

**Authors:** Qurratulann Alvi, Gul Muhammad Baloch, Karuthan Chinna, Ali Dabbagh

**Affiliations:** 1 School of Biosciences, Faculty of Health and Medical Sciences, Taylor’s University, Kualalampur, Selangor, 47500, Malaysia; 2 School Of Medicine,Faculty of Health and Medical Sciences, Taylor's University, Kualalampur, Selangor, 47500, Malaysia

**Keywords:** Ovarian Cancer, Reproductive Health, Lifestyle, Miscarriage, Pakistan

## Abstract

Ovarian cancer is a fatal gynaecological cancer and eighth most common cancer in women globally. Lifestyle, reproductive and sociodemographic factors are among the influential parameters that may significantly affect the risk of ovarian cancer and its mortality rate. However, the epidemiological investigations have shown that the risk of ovarian cancers associated with these factors is different in varied geographical distributions. Lifestyle and reproductive factors have not been investigated thoroughly across a wide cultural diversity. The objective of this study is to investigate the association of these factors with ovarian cancer in Pakistan. This investigation will focus on the lifestyle effects of fat intake, intake of tea, habitual exercise, use of talc, personal hygiene, habit of holding urine for long time, obesity on ovarian cancer among Pakistani women.  Reproductive variables will include age at menarche, natural menopausal age, parity, nulliparity (miscarriages, abortion, stillbirths), infertility, fertility treatment, tubal ligation, oral contraceptive use, and family history of breast or ovarian cancer. Sociodemographic variables will include effect of age, income, education, and geographical location. A case-control study will be conducted in the major cancer hospitals of Pakistan and the patients will also be interviewed. The controls will be recruited outside the hospital. For controls the same age limit and residency requirements will be applied. The information gained from this research will be an important contribution to develop programs for health promotion, with a focus on ovarian cancer prevention and women’s health. The findings could be used for health policies and planning to prevent ovarian cancer. The research will pave the way for a public policy and interventions to reduce the burden of ovarian cancer in Pakistan.

## Introduction

Ovarian cancer is one of the most frequently fatal gynaecologic cancers ( Jayson *et al.*, 2014; Tworoger & Huang, 2016). According to the American Institute for Cancer Research report, in 2018 ovarian cancer globally accounted for 3.6% of all forms of cancers and the eighth leading cause of death among women globally ( Merritt *et al.*, 2018). Only 46% of ovarian cancer patients survive beyond 5 years ( Kathawala *et al.*, 2018). The high mortality associated with ovarian cancer is due to late diagnosis and resistance to treatment, ( Carollo *et al.*, 2019; Nunes *et al.*, 2019).

According to a 2018 report by World Cancer Research Fund, the diagnosed cases of ovarian cancer are 295,414. The estimated age-standardized incidence and mortality rates of ovarian cancer in 2018 were 6.6 and 3.9, respectively ( Bray *et al.*, 2018) and this number is expected to reach at 434,184 by 2040.

The incidence, prevalence, and mortality of ovarian cancer varies across geographical locations and countries ( Coburn *et al.*, 2017). Globally, the highest incidence and mortality for ovarian cancer was reported in Serbia (16.6 and 6.8, respectively) and lowest in Gambia (0.6 and 0.43, respectively); in Europe itself, figures were highest in Serbia and lowest in Ireland (11.4 and 6.4, respectively); in Asia, rates are highest in Brunei (16 and 6.2, respectively) and lowest in Yemen (2.6 & 2.1, respectively) ( https://gco.iarc.fr/today/online-analysis-table).

The epidemiological diversity could be linked to different risk factors for ovarian cancer ( Hunn & Rodriguez, 2012). Poole *et al.* (2013) called for identifying modifiable risk factors. Jammal *et al.* (2017) believe that ovarian cancer prevention can be accomplished with clinical approaches.

The aetiology of ovarian cancer cannot be defined by single mechanism ( Terry & Missmer, 2017). Hence understanding of risk factors is important ( Webb & Jordan, 2017). Clinical evidence shows that by eliminating and decreasing the risk factors, the ovarian cancer cases can be prevented ( Bray *et al.*, 2018). Lifestyle modifications can minimize cancer burden ( Song & Giovannucci, 2016).

Ali (2018) noted that knowledge on ovarian cancer might increase survival rate. This can help women at risk of ovarian cancer to take special precautions ( Li *et al.*, 2015; Torre *et al.*, 2018); thus, help reduce ovarian cancer risk ( Momenimovahed *et al.*, 2019).

Schildkraut *et al.* (2019) reported obesity and family history of breast cancer to be major risk factors for ovarian cancer and Abbott *et al.* (2016) reported inadequacy of physical activity to be a risk factor. Gabriel *et al.* (2019) found the use of talc in genital areas to be a risk factor for ovarian cancer in England. In a Canadian study, Koushik *et al.* (2017) showed strong inverse association between parity and ovarian cancer. Harris *et al.* (2017) reported oral contraceptive and family history as the main risk factors in an American population. Lee *et al.* (2013) showed that consumption of green tea increases ovarian cancer risk in a Chinese population. Other risk factors include infertility ( Rasmussen *et al.*, 2017), miscarriages ( Moorman *et al.*, 2016) and age at menopause.

Doherty *et al.* (2017) suggests role of ethnically differing populations. Endpoints must represent the disease agent involved and rely on the quality of life ( Wilson *et al.*, 2017). Responsive and timely health care facilities that use relational communication, could enhance women's health experience with ovarian cancer ( Jelicic *et al.*, 2019).

### Study objective

This study explores lifestyle (use of talc, obesity, pattern of weight change), reproductive health (miscarriages, age at menarche, menopausal age, parity, nulliparity) and socio-demographic factors (age, income, and geographical location) associated with ovarian cancer in Pakistan.

## Methods

The research is now at an advanced stage after a development of a literature review, methodology and preparation of a validated questionnaire. The study will be conducted from July 2020 to December 2020. Data collection will start in July 2020.

### Study design

This will be a case-control study. The cases will be ovarian cancer patients registered at cancer hospitals in Pakistan. Hospitals are selected based on receiving approval from the hospital administrations. The controls will be recruited from the general population, using random digit dialling of individuals in the vicinity of the selected hospitals, since this was what the participating hospitals wanted. Cases and controls will not be matched. In the analysis, the outcome variables will be corrected for all the variables. We do not have the ability to mitigate many sources of bias as we must comply with the hospital’s’ requirements.

### Instrument

A validated questionnaire (validated by a panel of experts, each with doctorates and many years’ experience in biostatistics, public health and biomedicine) will be used to elicit information on following parameters.

1. Socio demographic characteristics

2. Patient clinical data

3. Diagnostic data

4. Lifestyle factors

5. Reproductive health

### Data collection

Patients’ clinical and diagnostic data will be obtained from the respective hospital registries. Written informed consent will be obtained from all participants with the voluntary decision to participate in the study. Interviews will be conducted with questions relevant to the parameters mentioned above.

### Sample size

Power and Sample Size software was used to calculate sample size. The level of significance and power were set as 0.05 and 80%, respectively. Based on results, the minimum required sample size is 387 for cases and 387 for controls. The figures are rounded up to 400 per group.

*Inclusion criteria for cases*

• Age 30 to 65

• Confirmed diagnosis of ovarian cancer by hospital or health care facility.

*Exclusion criteria for cases*

• Cognitively impaired

• Diagnosed with any other form of cancer

The inclusion criteria for control will be, age 30 to 65, healthy and cognitively not impaired; and exclusion criteria will be comorbidities, such as diabetes or cardiovascular disease.

### Data analysis

Data will be analysed using SPSS version 25. Qualitative variables will be described as frequencies and percentages, while quantitative variables will be described as means and standard deviations if the variable is normally distributed and as median and interquartile ranges, otherwise. Univariate and multivariate binary logistic regression analyses will be used in testing association between the predictor variables and ovarian cancer. In the univariate analysis, the predictor variables will be tested on at time. The variables that are significant at 0.25 level will be included in the multivariate analysis. Finally, stepwise analysis will be used to determine the significant predictors of ovarian cancer.

### Ethical consideration

Ethical approval for this study will be obtained from the participating hospitals/authorities in Pakistan. Respondents will be briefed, consent obtained and the confidentiality and participants’ right to stop at any point of the study will be assured.

## Data availability

No data are associated with this article.
