# Supplementary material for: Lifestyle and reproductive health: the aetiology of ovarian cancer in Pakistan
Source: F1000Res. 2020 Aug 4;9:901. [Version 1] doi: 10.12688/f1000research.24866.1 (PMC7417955; doi:10.12688/f1000research.24866.1)
Supplement: Peer review history

# Peer review: Reviewer response for version 1

**DOI:** 10.5256/f1000research.27437.r68704

**Published:** 2020-08-10

**Authors:** Sadiq Bhanbhro

**Affiliations:** 1 College of Health and Wellbeing and Life Sciences, Sheffield Hallam University, Sheffield, UK

This is a well-designed study protocol aimed to investigate the relationships between lifestyle factors, reproductive health, and risk of ovarian cancer in Pakistan. The aims, objectives and study design including sample size and data collection methods are appropriate and sufficiently described. The authors have cited the current and relevant literature. However, considering the sensitive nature of the topic under study, I would suggest the investigators need to provide more details on the recruitment of the potential study participants and the ethical considerations. For instance, the authors’ need to identify and name the relevant academic ethics review committee that will approve the project.

Is the study design appropriate for the research question?

Yes

Is the rationale for, and objectives of, the study clearly described?

Yes

Are sufficient details of the methods provided to allow replication by others?

Yes

Are the datasets clearly presented in a useable and accessible format?

Not applicable

Reviewer Expertise:

Public health, social sciences, research methods.

I confirm that I have read this submission and believe that I have an appropriate level of expertise to confirm that it is of an acceptable scientific standard.

# Peer review: Reviewer response for version 1

**DOI:** 10.5256/f1000research.27437.r68702

**Published:** 2020-08-07

**Authors:** Ramesh Kumar

**Affiliations:** 1 Health Services Academy, Ministry of National Health Services Regulation & Coordination, Government of Pakistan, Islamabad, Pakistan

This will be an important study proposal and will be helpful for future research evidence. Ovarian cancer is a big problem in Pakistan and researchers are strongly needed to work in this area. Moreover, the author has proposed a case-control study that would explore multiple factors responsible to develop this problem in Pakistan. 

 This is a study protocol developed by the author and the author will collect the data and publish it later on

Is the study design appropriate for the research question?

Yes

Is the rationale for, and objectives of, the study clearly described?

Yes

Are sufficient details of the methods provided to allow replication by others?

Yes

Are the datasets clearly presented in a useable and accessible format?

Yes

Reviewer Expertise:

Public Health

I confirm that I have read this submission and believe that I have an appropriate level of expertise to confirm that it is of an acceptable scientific standard.
